# Expert-Reviewed Nutritional Guidance for Adults with Spinal Cord Injury: A Delphi Study

**DOI:** 10.3390/nu17091520

**Published:** 2025-04-30

**Authors:** Ayse G. Zengul, Christine C. Ferguson, James H. Rimmer, Stacey S. Cofield, Elizabeth N. Davis, James O. Hill, Mohanraj Thirumalai

**Affiliations:** 1 Department of Nutrition Sciences, University of Alabama at Birmingham, Birmingham, AL 35233, USAkroeger@uab.edu (E.N.D.);; 2 UAB Research Collaborative, University of Alabama at Birmingham, Birmingham, AL 35209, USA; 3 Department of Occupational Therapy, School of Health Professions, University of Alabama at Birmingham, Birmingham, AL 35294, USA; 4 Department of Biostatistics, University of Alabama at Birmingham, Birmingham, AL 35294, USA; scofield@uab.edu; 5 SHP Research Collaborative, The University of Alabama at Birmingham, Birmingham, AL 35209, USA; 6 Division of Preventive Medicine, Heersink School of Medicine, The University of Alabama at Birmingham, Birmingham, AL 35233, USA

**Keywords:** spinal cord injury, nutrition recommendations, Delphi study, disability, dietary guidelines

## Abstract

**Background/Objectives:** Nutritional needs for people with chronic spinal cord injury (SCI) are inadequately addressed due to the lack of comprehensive evidence and scattered research. We established a consensus-based framework for addressing the nutritional needs of community-dwelling adults with chronic SCI who can ingest food orally. **Methods:** A web-based Delphi design was employed to ascertain an expert consensus. The Delphi panel consisted of physicians, registered dietitians (RDs), and researchers knowledgeable in SCI and nutrition. Informed by a literature review, 18 nutrition statements were rated by 15 panelists. The survey included statements about SCI-specific dietary energy assessments and macro- and micronutrients. **Results:** The response rate for the panel (N = 15) was 100%. Consensus levels, scores, stability levels, and response numbers were documented for each statement. The statements received consensus scores ranging from 4.14 to 8.13 on a 9-point Likert scale. Alternative expert comments and suggestions were also provided for each statement. **Conclusion:** Engaging a diverse panel of experts, the real-time Delphi process yielded expert-reviewed nutrition statements based on an extensive literature review and expert opinions. The rated statements contribute to the ongoing dialogue in SCI-specific nutrition, providing a practical resource for healthcare professionals working with adults with chronic SCI.

## 1. Introduction

Spinal cord injury (SCI) is a significant cause of long-term disability in the United States (US). The estimated number of individuals with SCI living in the US is approximately 299,000. There are approximately 18,000 new SCI cases each year [1,2].

Research on the nutritional needs of individuals with SCI is insufficient. Dietary recommendations intended for the general public are likely inappropriate, as they may be overestimated or underestimated due to the significant alterations in body composition associated with paralysis, neurogenic obesity, and obligatory myopenia, leading to depleted protein reserves, along with osteopenia, metabolic complications, a blunted anabolism, and reduced total energy expenditure [3,4,5]. The population with SCI often experiences a reduced resting metabolic rate (RMR) [6,7,8,9]., gut dysmotility [10,11], and sympathetic nervous system dysfunction [6,12], which contribute to neurogenic obesity and various cardiometabolic disorders, including insulin resistance, hypertension, dyslipidemia, and arteriosclerosis [3,4,13]. The Paralyzed Veterans of America (PVA) Consortium has published a clinical practice guideline for healthcare providers on the “Identification and Management of Cardiometabolic Risk after Spinal Cord Injury” [14]. However, this guideline primarily focuses on cardiovascular risk factors such as obesity, dyslipidemia, hypertension, and insulin resistance, incorporating dietary recommendations based on American Heart Association (AHA) guidelines. While these recommendations provide general dietary guidance, they do not fully account for the unique nutritional challenges faced by adults with SCI who consume food orally and live outside clinical or institutional settings. Therefore, there is a need for targeted nutrition guidance tailored to the specific dietary needs of adults with chronic SCI living in the community. This guidance should focus on key concerns such as SCI-specific energy prediction equations, micro- and macronutrient intake, nutritional supplements, and dietary factors to address the health problems commonly associated with SCI, such as constipation and pressure injuries.

The Advisory Committee of the Dietary Guidelines for Americans, 2020 (DGA) highlighted the need for nutritional guidelines for populations living with various disorders, including people with disabilities [15]. However, specific recommendations tailored to adults with chronic SCI remain limited. Health promotion programs established from evidence-based research are typically designed for the general public without disabilities, where greater resources are directed. Historically, individuals with disabilities are generally excluded from this research, resulting in a lack of reliable evidence specific to SCI [16,17]. At times when high-quality, reliable evidence is lacking, consensus-based methodologies offer a valid approach for drawing conclusions and developing recommendations to promote health and well-being [16,18]. Therefore, this study aims to summarize the existing literature and generate consensus-based nutrition statements for adults with chronic SCI living in the community, utilizing a web-based Delphi method. This study seeks to enhance dietary practice by offering updated and tailored nutritional guidance for this population, allowing experts to review and rate the best available knowledge to identify areas of consensus, despite the limited evidence. The resulting dissemination may serve as a valuable resource for health professionals and promote further research and collaboration.

## 2. Methods

### 2.1. Study Design

The nutrition statements were developed using an electronic Delphi methodology, a well-known and accepted technique recommended by the World Health Organization (WHO) to develop consensus-based guidelines [19]. Due to its practicality and cost-effectiveness, we used the online survey method, as the panel consisted of diverse health experts across the United States. We employed Calibrum Surveylet (Calibrum, Inc., St George, UT, USA), a well-regarded platform, for conducting the real-time Delphi method [20,21]. The web-based approach minimized common biases usually associated with face-to-face group interactions [22] and provided the anonymity, flexibility, and opportunity for reflection required by the study’s complexity.

### 2.2. Participant Selection and Panel Size

Experts from diverse professional backgrounds were recruited to avoid panel homogeneity. We utilized purposive sampling, a common approach in Delphi studies. While this method does not employ random selection, it ensures that panelists possess the specialized expertise necessary for informed consensus-building. The eligibility criteria included (1) a professional background as an RD, researcher with a doctoral degree, or physician and (2) a self-reported expertise in SCI and nutrition. Although there is no agreement on the sample size for Delphi studies [23], previous research suggests that panels typically range from 10 to 100 panelists [24]. The appropriate sample size depends on factors such as the complexity of the research question, available resources, and the level of expertise required [25]. If the samples are too small, the number of representatives may not be sufficient for a definite conclusion. Large sample sizes also have their drawbacks, such as low response rates and attrition [26]. Given the specificity of SCI nutrition expertise and our aim to maintain high engagement in the survey, we recruited 15 panelists to provide an informed consensus, while ensuring sustained participation.

### 2.3. Recruitment

Panelists were recruited through a strategic snowball strategy [27]. The experts were identified through networking within professional communities, referrals from recruited panelists, and a review of the published literature. Researchers were contacted if their publications were relevant to SCI and nutrition, and preference was given to those with multiple studies in this field, indicating specialized expertise.

### 2.4. Generation of Nutrition Statements

We systematically searched the PubMed, MEDLINE, and EMBASE databases to identify relevant studies investigating the nutritional needs and concerns of people in this population. We included terms such as ‘nutrition’ and ‘spinal cord injury’ and utilized advanced indexing features (e.g., ‘exp’ in EMBASE) for precise indexing. The identified publications were uploaded to EndNote X9 (Clarivate, Philadelphia, PA, USA) for title screening. NVivo software(QSR International, 2018, NVivo 12) was used for a full-text screening and qualitative review of the literature. The inclusion criteria prioritized peer-reviewed articles and systematic reviews in English, focused exclusively on human studies, excluding animal research, without imposing any specific time restrictions. The relevant references in the identified articles were also examined to generate statements. The data extraction sheet covered energy expenditure, anthropometry, micro- and macronutrient intake, water and alcohol consumption, and nutrition-related symptoms associated with injury level and diet type. Based on the extracted data, a draft set of nutrition statements was created by the research team. After completing the initial draft of the statements, three specialists in either SCI or nutrition (JH, CF, and CY) reviewed and refined it, before finalizing the content for the Delphi survey (Table 1).

### 2.5. Delphi Procedure and Data Analysis

The Delphi panel consisted of dietitians, researchers, and physicians from various professional backgrounds. Panelists were invited to the Surveylet platform via email. A detailed introduction and instructions regarding the Delphi study procedures were provided for the participants. The demographics of the panelists were collected (Table 2). Reminder emails were sent following the initial invitation to increase participation. In addition to the statements generated through the literature review, the survey included several open questions to identify any additional recommendations or aspects that may have been overlooked. The panelists were asked to rate the statements based on their importance and/or usefulness, considering how critical each statement was and how feasible it would be to implement in real-world settings. They rated their level of agreement with each statement using a 9-point Likert scale, with higher scores indicating stronger consensus. A 9-point scale was selected because it allows for a broader range of responses and enhances differentiation between levels of agreement. A comment box was included with each statement to allow panelists to provide feedback on the statements. Also, an alternative text box was provided for those who wished to suggest modifications or propose new recommendations. These alternative suggestions were then presented to the panel for rating and comment. The Delphi process was conducted asynchronously, allowing panelists to complete evaluations at their convenience. After submitting their ratings, panelists could view other panelists’ ratings in real time, helping to facilitate a faster consensus. Additionally, a real-time statistical summary was provided for each statement. Panelists had the opportunity to revise their responses based on the aggregated results, allowing for iterative refinement of the statements. Since the Delphi survey was conducted online with no fixed rounds, once all panelists had initially submitted their responses, we sent follow-up email invitations encouraging them to review comments, consider suggestions, and make any revisions if needed. The ratings of the panelists and the consensus details for each statement are presented. The ratings were categorized as follows: 1–3 (poor agreement), 4–6 (moderate agreement), and 7–9 (high agreement).

### 2.6. Anonymity and Confidentiality

Each panelist was assigned a unique identification code to maintain anonymity and protect confidentiality. The Calibrum (Surveylet) platform complies with the General Data Protection Regulation (GDPR). Written informed consent was required before participation. Study procedures were approved by the UAB Institutional Review Board (IRB).

## 3. Results

A response rate of 100% was achieved, with 15 experts actively participating in the web-based real-time Delphi survey on nutrition statements for people with SCI. The expert panel comprised seven RDs, two physicians, and six researchers with experience in the SCI and nutrition fields. Most panelists held a doctorate or professional degree and had varying levels of experience in SCI nutrition. While several panelists had relatively fewer years of experience, they were invited for their specialized knowledge in the nutrition and SCI fields. Panelists demonstrated moderate to high familiarity with the SCI-related nutrition literature, with a mean score of 7.26 (standard deviation 1.41) on a scale of 1 to 10. Detailed demographic characteristics are presented in Table 2.

### Summary of the Nutrition Statements for Adults with Chronic Spinal Cord Injury (SCI) Who Can Consume Food Orally and Live Outside of Clinical or Institutional Settings

Each statement is accompanied by a literature summary and its consensus scores, reflecting the level of agreement among panelists. Statements 1–6 suggest alternative methods for energy calculation where indirect calorimetry is not available or applicable.

Statement 1—Persons with paraplegia should weigh 5–10% less than indicated in the Metropolitan Life Height–Weight Table. Persons with tetraplegia should weigh 10–15% less than indicated in the Metropolitan Life Height–Weight Table. (Consensus score = 4.14)

Although standard obesity classifications like BMI may not fully apply to individuals with SCI [29], according to the Academy of Nutrition and Dietetics Spinal Cord Injury Guideline [30], there is evidence that a 5–10% and a 10–15% reduction in the Metropolitan Life Height–Weight Table weight should be used for paraplegia and tetraplegia, respectively, to adjust the ideal weight for people with SCI.

In the Metropolitan Life Height–Weight Table, ideal body weight is determined based on the frame size of the person. Body frame size is determined by using wrist circumference in relation to the person’s height and gender (see Table 3) [31,32,33,34]. Still, the Metropolitan Life Height–Weight Table has not previously been validated in a SCI population.

However, the Metropolitan Life Height–Weight Table only covers the ages between 25 and 59 years. Therefore, some studies [35,36] have utilized the Hamwi equation [37,38] to identify the ideal body weight in older populations with SCI; they then incorporated the weight reductions suggested by the Academy of Nutrition and Dietetics Spinal Cord Injury Guideline [30]. The Hamwi formula uses height and sex information to calculate the ideal body weight. According to the formula, a male’s ideal body weight is calculated as 106 pounds for the first 5 feet, plus 6 pounds for each additional inch. For females, the ideal body weight is calculated as 100 pounds for the first 5 feet, plus 5 pounds for each additional inch.

Statement 2—Individuals with SCI whose body mass index (BMI) is 22 kg/m^2^ or greater should be classified as having obesity. (Consensus score = 5.47.)

Although BMI is often utilized for individuals with SCI, the measure and its weight categories underestimate obesity because it cannot differentiate specific tissue types related to body composition [8,9]. People with SCI generally have a higher fat mass coupled with a lower lean body mass for a given BMI due to muscle atrophy [39,40,41]. Based on strong evidence, the PVA Consortium for Spinal Cord Medicine expert panel [14] recommended classifying adult women with >35% body fat (BF) and adult men with >22% BF as obese. According to the consortium, BMI cut-offs > 22 kg/m^2^ should be used to define obesity. Adults with SCI, both men and women, with a BMI > 22 kg/m^2^ should be considered at high risk for cardiometabolic disease (CMD) [3,14,42,43]. Similarly, the Study of Health and Activity in People with Spinal Cord Injury (SHAPE-SCI) research group conducted a study involving 77 adults with chronic SCI who underwent anthropometric measures, and it concluded that BMI cutoffs for the general population fail to identify obesity in 73.9% of adults with SCI. The study also suggested using a BMI > 22 kg/m^2^ cutoff point as an indicator for obesity and obesity-related chronic diseases [39].

Statement 3—Waist circumference: The optimal cutoff should be 86.5 cm in men with SCI (Consensus score = 5.20.).

Waist circumference, waist-to-height ratio, waist-to-hip ratio, and neck circumference have been utilized to identify obesity in the general population. Among those, waist circumference and waist-to-height ratio are strongly correlated with adiposity and cardiovascular disease (CVD) risk scores [44,45]. Considering the challenges in measuring height in people with SCI, waist circumference provides a more practical assessment of obesity-related CVD risk in this population.

In the general population, waist circumference is strongly associated with CVD risk factors [46,47,48] as being a strong predictor for total BF and visceral adipose tissue [46,49,50,51]. Similarly, several studies indicated strong correlations between waist circumference and CVD risk factors in the SCI population [52,53,54]. However, for a given waist circumference, individuals with SCI have greater visceral adipose tissue than able-bodied individuals, indicating the need to adjust the waist circumference cutoff for obesity in individuals with SCI [45,55].

In 2018, Sumrell et al. proposed SCI-specific waist circumference cutoffs to identify persons who are at risk of CVD based on an MRI-VAT_CSA_ (Magnetic Resonance Imaging–Visceral Adipose Tissue Cross-Sectional Area) of greater or less than 100 cm^2^ [56,57]. The study proposed an 86.5 cm cutoff for supine waist circumference and an 88.3 cm cutoff for abdominal circumference [56].

In the general population without SCI, waist circumference is measured in a standing position. However, abdominal muscle tension may be impaired in people with SCI. While both seated and supine measurements of waist circumference have been assessed, the supine position may provide a more consistent measurement due to impaired muscle tension, as described by Sumrell et al. [41]. In their study, waist and abdominal circumferences were measured by an inflexible tape measure in sitting and supine positions after exhalation. Waist circumference was assessed at the midpoint between the lowest rib margin and the crest of the ilium. The results of several studies that applied the SCI-specific cutoff supported the study by Sumrell et al. and suggested the usage of an 86.5 cm cutoff to identify individuals at risk for developing CVD, obesity, and cardiometabolic syndrome, although the validity of this cutoff is still debated in the literature [57,58,59]. Notably, only male participants were included in this study.

Statement 4—In applicable cases, the SCI-specific energy prediction equation proposed by Chun et al. [28] should be used (BMR = 24.5 × FatFreeMass + 244.4). (Consensus score = 5.07)

In predicting the basal metabolic rate (BMR) for people with SCI, the existing equations developed for people without disabilities show a reduced accuracy [60,61,62,63]. People with SCI have a lower basal metabolic rate compared to people without disabilities [61,64,65,66]. For example, the equations by Harris–Benedict [67] and Schofield et al. [68] over-predict the BMR by 15–32% and 6%, respectively [66]. The literature indicates that adjusting for body mass or fat-free mass improves the accuracy of the equations [64]. Buchholz et al. [9], Chun et al. [28], and Nightingale and Gorgey [66] developed SCI-specific prediction equations for estimating metabolic rate [61]. Each study measured fat-free mass by dual-energy X-ray absorptiometry (DXA). In contrast, the equation formulated by Chun et al. was cross-validated to determine the accuracy of the existing metabolic rate prediction equations in men with chronic SCI [66]. The study involved East Asian participants who had a notably lower mean fat-free mass than participants in other studies [66]. Nevertheless, this equation demonstrated the lowest mean ± SD bias (1 ± 6%; 3 ± 91 kcal/day) among the equations tested. Additionally, unlike the equation developed by Nightingale and Gorgey et al. [66], Chun et al. [28] developed the equation for both genders.

Statement 5—In the absence of fat-free-mass information, the Mifflin–St Jeor equation should be used to predict the RMR in persons with chronic SCI. (Consensus score = 4.40)

Due to muscle atrophy, individuals with SCI have a reduced fat-free mass and greater fat mass for a given BMI compared to individuals without SCI [39,40,41]. DXA provides a simpler assessment of fat-free mass compared to other laboratory tools such as MRI, bioelectrical impedance, ultrasound imaging, and intracellular body K ⫹ (potassium ion) [69]. These tools are often unavailable due to time, cost, and the need for extensive training [69]. Therefore, in the absence of clinical tools, direct analyses of body composition, or indirect calorimetry, Nightingale and Gorgey et al. suggested using anthropometric measurements for predicting the RMR in the SCI population [66]. This suggestion is in accordance with data from studies that included individuals without disabilities, which indicates that anthropometric data present a helpful alternative resource to predict the RMR when details about body composition are not available [66,70]. Moreover, Broad et al. measured the RMR in wheelchair rugby athletes, reporting an RMR of 1735 ± 257 kcal per day [71]. This result supports the statistical similarity of equations by Chun et al. [28], Nightingale and Gorgey et al. [66], Cunningham et al. [72], Mifflin et al. [73], and Owen et al. [74] as an alternative to measure the RMR [61,71]. The Mifflin–St Jeor equation uses body weight, height, age, and sex, making it practical for non-clinical settings [73]. Similarly, Pelly et al. [75] compared RMR prediction equations and reported no significant difference between the measured RMR for athletes with SCI and the predicted RMR from the Mifflin–St Jeor [73], Cunningham [72], Schofield [68], and Harris and Benedict [67] equations. They also reported that the model formulated by Mifflin et al. showed the most robust relationship to the measured RMR across five regression models, including Owen et al. [74]. Various studies conducted on different populations also found that the Mifflin–St Jeor equation demonstrated the highest reliability in predicting the RMR [76,77,78,79,80]. For further details, see Table 4.

Statement 6—In the population with chronic SCI, the total daily energy expenditure (TDEE) should be estimated by multiplying the basal metabolic rate (BMR) with the common activity correction factor of 1.15. TDEE: BMR X 1.15. (Consensus score = 6.60)

TDEE is estimated based on a person’s physical activity, metabolic rate, and the thermic effect of food (TEF) [83]. SCI leads to blunted sympathetic nervous system responses, a decreased dietary composition and energy intake, and increased adipose tissue [84,85]. Consequently, the TEF is reduced following SCI. Although the TEF corresponds to only 8–10% of TDEE, decreased energy expenditure can result in a significant increase in adipose tissue over time [84]. In the absence of indirect calorimetry or doubly labeled water, the Academy of Nutrition and Dietetics recommends using the Harris–Benedict formula, which utilizes weight, an injury factor of 1.2, and an activity factor of 1.1 [30,86]. However, this prediction model is not SCI-specific and overestimates TDEE and energy intake in people with SCI, leading to overfeeding [65,87,88]. Recently, a novel SCI-specific correction factor of 1.15 was developed by Farkas et al. According to this equation, after estimating a person’s BMR, the value will be multiplied by 1.15 to account for the decreased energy needs associated with SCI. This correction factor in estimating TDEE was determined using the metabolic rate, 2.7 mL of oxygen/kg of body weight/min [89], and a metabolic equivalent of task (MET) in persons with SCI [83].

One MET is equal to the energy expenditure in quiet sitting and is defined as a resting oxygen consumption of 3.5 mL·kg^−1^·min^−1^ for people without disabilities. However, a 23% lower resting oxygen consumption has been reported in people with SCI using indirect calorimetry. Persons sustaining upper-level SCI displayed marginally reduced resting values at 2.52 ± 0.50 mL·kg^−1^·min^−1^ compared to 2.77 ± 0.47 mL·kg^−1^·min^−1^ for those with lower-level SCI. This difference between the two injury-level groups was not significant [89]. Collins et al. compiled energy expenditure values for physical activities among individuals with SCI. The authors determined that 1 MET corresponded to 2.7 mL·kg^−1^·min^−1^ for individuals with SCI. They concluded that utilizing a metabolic equivalent of 3.5 mL·kg^−1^·min^−1^ may result in an underestimation of the intensity level of their physical activity [89]. Similarly, several studies have reported a lower BMR in those with SCI [6,7,82,90]. Farkas et al. used a MET level of 2.7 mL·kg^−1^·min^−1^ to develop the SCI-specific activity correction factor of 1.15 [83].

In a meta-analysis, the authors demonstrated that in a sample of 606 individuals with chronic SCI, the pooled resting metabolic rate was 1492 kcal/day, while the total energy intake was 1876 kcal/day. By using the equation of BMR × 1.15, the authors reported a positive energy balance, indicating overeating [8]. In “Cardiometabolic Disease and Dysfunction Following Spinal Cord Injury: Origins and Guideline-Based Countermeasures”, Nash and Gater also recommended using the correction factor of 1.15 because of the alterations in body composition, besides the adrenergic and metabolic differences associated with SCI [84]. By estimating the SCI-specific TDEE, a negative energy balance can be attained by employing a calorie reduction and/or increased physical activity [3].

Additionally, while there may not be specific evidence and strong guidelines available for estimating the energy needs of athletes with SCI or regularly active individuals (i.e., exercisers) with SCI, the equation TDEE = BMR × 1.15 may still be applicable. This equation considers BMR/RMR, which factors in metabolically active tissue when measured or estimates it based on body weight when predicted [83,89]. Therefore, it may be used to estimate the energy needs of athletes with SCI or regularly active individuals with SCI.

Statement 7—Carbohydrate intake should be 45% of the total daily energy intake for people with SCI. (Consensus score = 5.73)

The Dietary Guidelines for Americans [91], Institute of Medicine [92], and Australian Dietary Guidelines [93] recommend 45–65% of the total daily energy intake from carbohydrates [65,94]. According to Farkas et al., the DGA recommendations of 45 to 65% carbohydrate intake may not be appropriate for individuals with SCI. Additionally, these recommendations do not account for the physiological and anatomical changes caused by neurological injury [4]. The extensive muscle atrophy in people with SCI impacts the primary carbohydrate storage site and leads to increased circulating plasma glucose and, eventually, the development of type-2 diabetes mellitus. Also, reduced physical activity impacts the utilization of glucose and fat and expands the adipose tissue sites [95]. Several studies suggest that consuming 45% of total daily calories from carbohydrates may help maintain their body composition and cardiometabolic health in people with chronic SCI [4,65,95,96]. Enteral diets in SCI traditionally promote 45% carbohydrate nutritional content, whether the care is acute or sub-acute [97,98]. However, this suggestion requires further investigation.

Statement 8—Total dietary fiber intake should be 15 to 20 g a day, with adequate fluid intake, for people with SCI (Consensus score = 6.47.).

The Dietary Guidelines for Americans (DGA) recommends consuming 22 to 34 g/day of fiber [4]. However, the Academy of Nutrition and Dietetics Evidence Analysis Library (ANDEAL) recommends 15 g/day of fiber and notes that a fiber intake higher than 20 g per day may lead to prolonged intestinal transit times in people with SCI [30]. Supporting this recommendation, Cameron et al. investigated the influence of increased fiber intake on large bowel function and suggested that a high dietary fiber intake (>20 g/day) does not have an equivalent effect on bowel function in people with SCI as in healthy individuals [99]. A high fiber intake may be associated with increased transit time, fecal impaction, irregular stool, and chronic constipation in the SCI population [30,99]. A study by Badiali et al. concluded that around 15 g/d of dietary fiber intake could improve the neurogenic bowel function in SCI, whereas a fiber intake greater than 25 g/d would increase transit and evacuation times [100,101]. Thus, several studies suggested that high-fiber diets would be impractical for persons with SCI [4,43,65,95,99,100,102,103]. Furthermore, a high fiber consumption would require an adequate fluid intake to prevent constipation [10]. Excess fluid intake, on the other hand, may lead to bowel/bladder accidents or require additional urinary catheterization [43,65]. Although a minimum of 1.5 L fluid intake is recommended, further research is needed to address this point [95].

Statement 9—Individuals with SCI need 0.8 to 1.0 g of protein per kg of body weight, or roughly 10–30% of daily caloric intake, to maintain protein stores in the absence of pressure injuries or infection. (Consensus score = 5.40)

An adequate protein intake is necessary to preserve muscle mass and prevent pressure injuries. The evidence-based nutrition practice guidelines published by the National Pressure Ulcer Advisory Panel (NPUAP), the European Pressure Ulcer Advisory Panel (EPUAP) [104], and the Academy of Nutrition and Dietetics [30] provide basic protein recommendations for people with SCI for both acute and rehabilitation phases. However, the evidence is rated as weak, and more research is required to determine the proper protein requirements. Based on the current literature, the protein requirements for people with SCI align with the general nutritional guidelines, with the exception of cases where a pressure injury is present [4,5,30,95,105]. Several studies indicated that protein consumption in people with SCI is within or exceeds the recommended USDA values [4,8,55,94,103,106,107,108,109,110,111,112,113,114,115,116,117,118]. However, although protein consumption is usually high among people with SCI, some individuals may still not meet the dietary recommendations for specific amino acids, including lysine, leucine, cysteine, threonine, and methionine [8,65,109,119]. This may result in malnutrition and other health issues, as these amino acids are essential for protein synthesis and tissue repair [65].

Statement 10—Protein recommendations for individuals with SCI who have pressure injuries are as follows:

Stage II: 1.2 to 1.5 g of protein per kg body weight/per day.

Stage III and IV: 1.5 to 2.0 g of protein per kg body weight/per day. (Consensus score = 6.87)

According to the Academy of Nutrition and Dietetics Evidence Analysis Library (ANDEAL), there is a consensus that additional protein consumption is required for the optimal healing of pressure injuries [30,95]. In these instances, protein needs can be calculated as stated above. The PROT-AGE Study Group evidence-based guideline suggests that “those with severe illness or injury may need 2.0 g/kg body weight per day” [104,120]. Also, severe pressure injuries may lead to increased fluid loss due to the evaporation of fluids, fever, or air-fluidized beds. Therefore, the dietitian must monitor the parameters of hydration status. According to evidence-based guidelines, daily fluid intake requirements can be estimated as 1 mL/kilocalorie consumed daily [121]. However, individuals experiencing conditions such as an elevated body temperature, profuse sweating, vomiting, diarrhea, and/or heavily exuding wounds often require an additional fluid intake to compensate for fluid loss. Individuals adhering to high-protein dietary regimens may also require an additional fluid intake [104,121,122].

Statement 11—For individuals with SCI who have developed a pressure injury, dietitians should recommend the use of daily mineral and vitamin supplements within recommended dietary allowance (RDA) levels. (Consensus score = 7.33)

Certain micronutrients, such as vitamin A, vitamin C, iron, and zinc play, a role in wound healing. The dietitian may consider recommending the relevant mineral and vitamin supplements with caution [30,105]. No comprehensive evidence-based practice guideline has been developed for the micronutrient needs of this population. However, due to safety concerns related to excessive intake, it is recommended that if micronutrient supplementation is indicated in the presence of a pressure injury, it should not exceed 100% of the RDA. The need for micronutrient supplementation should be re-evaluated every 7 to 10 days [30]. It is important to exercise caution when supplementing above the Tolerable Upper Intake Level (UL).

Vitamin A

Vitamin A deficiency can adversely affect wound healing. It can disturb the immune function and elevate the risk of wound-related infections. The Tolerable Upper Intake Level (UL) for vitamin A is 3000 mcg (10,000 IU) per day/day. However, moderately–severely injured patients or malnourished patients may be recommended a 10,000 IU IV for a limit of 10 days. If the patients are under steroid treatment, 10,000 to 15,000 IU of vitamin A supplementation is recommended for one week to attenuate the anti-inflammatory effects of the steroids. The recommendations should be provided cautiously and judiciously due to the potential toxicity of vitamin A supplementation [30].

Vitamin C

Vitamin C exerts a vital role in wound healing. Supplementation demonstrated an improvement in wound healing among people with a vitamin C deficiency [105]. High doses of vitamin C supplementation are commonly recommended in the literature for healing chronic wounds. The Agency for Health Care Research and Quality, for example, suggests 100 to 200 mg of daily vitamin C for individuals who have stage I and II pressure injuries and 1000 to 2000 mg for stage III and IV pressure injuries [30,123].

Zinc

Zinc deficiency can lead to decreased collagen and protein synthesis, which is associated with delayed wound healing due to impaired immune competence. The recommendation for adults is 50 mg elemental zinc (ZnSo4 220 mg) twice per day. High-dose zinc supplementation should be provided for no more than two to three weeks. The dosage of zinc supplementation should be personalized based on zinc status and the metabolic demands of the individual [30,123].

Iron

Anemia is associated with poor wound healing due to a reduced oxygen supply in tissues. When hemoglobin and hematocrit concentrations are low due to iron-deficiency anemia, it may be associated with tissue hypoxia and impaired wound healing [30,105]. Therefore, iron supplementation should be recommended, as indicated in RDA suggestions (8 mg/day for men and 18 mg/day for women- 19–50 years old), to correct iron-deficiency anemia [30,124].

Statement 12—For those with SCI, total fat should be ≤30% and saturated fat ≤ 5–6% of daily calories. (Consensus score = 5.93)

The literature indicates that fat intake in individuals with SCI is usually above the recommended level of 30% [102,108,109,111,113,116,125,126,127]. An excessive fat intake may lead to various health problems, including metabolic syndrome associated with obesity, high triglycerides, high blood pressure, and insulin resistance [95,128,129]. In the DGA [91], the recommended intake is 20–35% of total fat, of which less than 10% comes from saturated fat. However, the World Health Organization (WHO) recommends reducing the total fat intake to no more than 30% of the total energy intake to maintain a healthy diet [130]. Similarly, the WHO [130], Public Health England (PHE) [131], and Australian Dietary Guidelines (ADG) [93] suggest limiting saturated fat intake to <10–11% of the daily energy intake. However, the American Heart Association (AHA) [132,133,134] recommends limiting it to 7%, while the Institute of Medicine (IOM) [92] suggested reducing it to as low as possible [65]. However, for persons with SCI, the Paralyzed Veterans of America Clinical Practice (PVA) guideline recommends limiting saturated fat intake to 5–6% of the total energy intake because of the body composition alterations and the significant reduction in energy expenditure and energy needs after SCI [14,65].

Statement 13—People with SCI must consume adequate micronutrients based on the Dietary Guidelines for Americans (DGA). (Consensus score = 6.67)

Micronutrients are involved in various metabolic functions and are vital to healthy development, disease prevention, and well-being. In the SCI population, vitamins such as A, B5, B7, B9, D, E, and C, and minerals including calcium, chloride, magnesium, and potassium are reported below the daily recommended intake based on the DGA [8,43,102,103,135,136,137]. Some of those micronutrients are essential in preventing and treating symptoms in persons with SCI. Deficiencies in these vitamins and minerals might be associated with impaired glucose [138,139] or lipid metabolism [102,140,141,142]. Vitamin D intake, for example, may impact cholesterol and carbohydrate profiles by decreasing total cholesterol [143,144,145,146,147,148] and improving glucose homeostasis [149,150,151,152,153,154,155,156,157,158,159,160]. The correlation between obesity and vitamin D deficiency is also well documented [118,149,157,161,162,163,164]. Vitamin D deficiency is more prevalent among overweight and obese individuals [161]. Adequate vitamin D intake is also important in pressure injury prevention [165]. Zhou et al. reported a correlation between vitamin D status and the occurrence of pressure injuries [166]. According to the study, people without pressure injuries have significantly higher vitamin D concentrations than those in the SCI population. Similarly, zinc levels are shown to be lower in persons with SCI who have pressure injuries. Zinc is essential in collagen production and cell repair [167,168]. Zinc deficiency can reduce collagen synthesis, which has a critical role in regulating wound healing [169]. Vitamin B and C deficiencies are also linked to impaired wound healing and anemia [170], which are highly prevalent after SCI [171,172]. According to several studies, lower levels of vitamin C [173] and magnesium [138] are also associated with existing atherosclerosis and diabetes. Moreover, calcium and vitamin D are important for bone metabolism, considering the high prevalence of osteoporosis and osteopenia in this population [65,174]. Therefore, people with SCI must prioritize making optimal dietary choices to ensure that they consume adequate amounts of vitamins and minerals as outlined by the USDA dietary guidelines, ideally sourced from nutrient-dense food options, to reduce the risk of inadequate nutrient consumption.

Statement 14—People with SCI should receive dietary supplements with caution and only if certain deficiencies are detected or to prevent them. (Consensus score = 8.13)

Micronutrient supplements are common among people with SCI [103]. The literature indicates some benefits of dietary supplements, such as docosahexaenoic acid-rich fish oil (DHA-FO) and vitamin D, on various symptoms related to SCI [118,175]. Marques et al. reported that DHA-rich fish oil supplementation could mitigate the markers of muscle damage and alterations in the lipid profile and inflammatory mediator levels, as well as suppressing the neutrophil death caused by acute exercise in wheelchair athletes [175]. Also, vitamin D supplementation has shown potential benefits for wound healing in patients with pressure injuries [165]. Although dietary supplementation may offer benefits for wheelchair athletes and improve specific deficiencies, people with SCI should be prescribed them judiciously, especially if there is no detected deficiency. A greater emphasis should be placed on consuming a healthy diet that includes adequate micronutrients, rather than relying on supplements, to minimize toxicity, interactions with prescribed medications, and other health-related risks [65].

Statement 15—Fluid intake should be adjusted based on health condition, age, sex, climate, and physical activity level. (Consensus score = 7.73)

Daily fluid intake refers to the amount of water consumed from plain water, foods, and other beverages [176]. There is no specific recommendation for fluid intake [176,177]. The recommendations for water consumption should be adjusted depending on personal health conditions, activity level, and environmental circumstances [176,177]. The symptoms of neurogenic bowel dysfunction, including constipation and fecal incontinence, frequently occur in people with SCI [178]. Water intake is especially important for bowel dysfunction symptom management [43]. People with SCI and neurogenic bowel dysfunction often experience an increase in colonic transit time, which leads to excessive fluid reabsorption and hardened stools [30]. A high fiber consumption without adequate fluid intake can result in constipation [10]. However, fiber intake with excess fluid intake may also lead to bowel/bladder accidents or necessitate the use of a urinary catheter [43]. On the other hand, some foods and beverages, including caffeine, alcohol, coffee, tea, cola, chocolate, figs, prunes, and sorbitol-containing foods, can result in the overstimulation of bowel activity and watery stools [179]. Adequate water consumption is necessary for overall health. Although the total fluid intake can come from various foods and beverages, plain drinking water is a good option for obtaining adequate fluids, as it has no calories [176]. Drinking plain water can help with managing body weight, reducing caloric intake [180,181,182], and preventing dehydration, which is a condition that can lead to mood changes, unclear thinking, an overheated body, constipation, and kidney stones [183,184]. If the person has a pressure injury, fluid loss might occur due to evaporation from wound drainage, open wounds, and fever; therefore, the person’s hydration status should be assessed to determine their fluid needs [30]. According to the Academy of Nutrition and Dietetics, current daily fluid recommendations for the non-SCI population are 30 to 40 mL/kg or a minimum of 1.0 mL/kcal per day. Additional fluids may be required based on personal conditions [30].

Statement 16—Sodium consumption should be ≤2400 mg/d for people with SCI. (Consensus score = 6.47)

Most sodium in a diet comes from added salt. Reducing sodium consumption lowers the risk of high blood pressure, stroke, and heart disease [185]. The 2020–2025 Dietary Guidelines for Americans recommend consuming less than 2300 mg of sodium per day to maintain a healthy eating pattern [91]. Conversely, the sodium intake by people with SCI usually exceeds 2300 mg [103,108,109,116]. Given the elevated sodium consumption and prevalence of hypertension among people with SCI [186,187], the Paralyzed Veterans of America (PVA) Consortium guideline recommends a sodium consumption ≤ 2400 mg/d for all individuals with SCI [14]. Moreover, some people with SCI may experience functional sympathectomy and consequently suffer from hypotension [41,109,188,189,190,191,192]. In those individuals, personalized considerations (e.g., lowering the sodium intake to ~1500 mg/day based on the injury level, focusing on whole foods, and monitoring blood pressure regularly) should be made when recommending sodium consumption [102].

Statement 17—Alcoholic beverages should be avoided by individuals with SCI. (Consensus score = 5.80)

In individuals with SCI, the use of alcohol has been linked to increased medical problems [43,193,194,195,196,197]. Alcoholic drinks can potentially interact with phytochemicals and drugs and affect bowel motility, energy, and water balances [43,193]. Therefore, alcoholic beverages should be avoided by people with SCI. However, if a person chooses to consume alcohol, the American Diabetes Association [198] and the 2020–2025 DGA [91] recommend limiting alcohol intake to one drink or less in a day for women and two drinks or less in a day for men. One alcoholic beverage is usually defined as a 5 oz. glass of wine, a 12 oz. beer, or a 1.5 oz. glass of 80-proof spirits, each containing approximately 15 g of alcohol [198,199].

Statement 18—The DASH or the Mediterranean diet can be adopted by individuals with SCI if hypertension or other cardiometabolic risk factors are present. (Consensus score = 6.93)

CVDs are a growing concern among individuals with SCI [200,201,202]. Despite the relatively young age of many individuals with SCI, their compromised metabolic condition often places them at an elevated risk for CVD events [203,204]. The major modifiable cardiovascular risk factors for chronic SCI patients include physical immobility, irregular blood pressure, glycemic control, dyslipidemia, and chronic inflammation [203,205,206,207,208,209]. Blood pressure irregularities could contribute to vascular problems and lead to a greater risk of arterial disease in people with SCI [210]. Adopting a heart-healthy dietary approach focusing on fruits, vegetables, whole grains, poultry, low-fat dairy, legumes, fish, and nuts, while limiting red meat, saturated fat, and added sugar, would help prevent cardiovascular problems in this population [3,14]. According to the literature and the Paralyzed Veterans of America (PVA) Consortium for Spinal Cord Medicine expert panel [14], the DASH (Dietary Approaches to Stop Hypertension) and/or Mediterranean dietary pattern may be recommended to help reduce inflammation and other cardiovascular risk factors in individuals with SCI [5,14,211,212].

Additional information is provided in Table 5, which summarizes the consensus ratings, scores, responses, and stability measures for each statement. The consensus status indicates whether the panelists agreed on a statement. The score indicates the mean rating on a scale of 1 to 10. Responses represent the number of panelists who provided ratings for each statement. Stability measures the level of agreement among the panelists, with consensus attainment determined using a threshold of 50% [213] as per the Surveylet platform (Calibrum). This threshold is used to compute and display the results, allowing readers to interpret the consensus scores provided for each statement. Moreover, the comments and suggestions of the experts for the statements are reported in Table 6. The panelists’ consensus status and response numbers are included to provide further context or clarification for each comment.

## 4. Discussion

This Delphi study provides expert-reviewed nutritional guidance for adults with SCI. This study fills a critical gap in the current literature, offering a consensus-based framework that can be used in the dietary management of this population. Individuals with SCI face unique challenges and requirements related to macro- and micronutrients and energy intake, highlighting the importance of tailored and appropriate nutrition recommendations to support their overall well-being. Therefore, we aimed to bridge the gap between the limited availability of strong evidence in this area and the pressing need for practical nutrition recommendations for thousands of individuals living with SCI. While acknowledging the restricted availability of robust evidence [4], our study contributes to ongoing research by synthesizing the existing literature and providing valuable insights.

To understand the current advice, we conducted a literature review, meticulously compiling the available evidence to generate a set of evidence-based nutrition statements. Although the most accurate means of estimating energy expenditure for individuals with SCI involve utilizing indirect calorimetry or some other clinical devices [216], we recognize that access to such equipment may be limited in non-clinical settings [69]. Therefore, the insights we present aim to be practical and accessible, offering workable solutions when specialized clinical devices are unavailable.

Individuals with SCI have distinct nutritional needs due to the associated and secondary conditions that accommodate their disability [3,4,5,217]. Predictive equations often prove inadequate for this population, thus requiring research to explore alternative solutions. Modifying metrics such as BMI, waist circumference, metabolic rate, and daily energy expenditure equations seeks to enhance the accuracy and reliability of the estimated energy requirement calculations [69,83]. Precision in determining the energy requirements of people with SCI would enable the formulation of more tailored recommendations concerning optimal nutrient and caloric intake. This matter is particularly important due to disability-related conditions such as lower physical activity levels, increased susceptibility to pressure injuries, and the prevalence of bowel problems and constipation associated with diet [95,99]. Our Delphi study systematically incorporated the available evidence and expert guidance to establish nutrition recommendations for adults with chronic SCI.

In addition to macronutrient considerations, the Delphi survey included various statements related to micronutrients. The results encompass consensus scores and alternative suggestions and comments for each recommendation. These alternative suggestions and comments, followed by ratings from the panelists, aimed to enrich the discourse and provide a comprehensive exploration of expert opinions and discussions beyond numerical scores by enabling readers to explore not only the degree of consensus but also the panelists’ alternative viewpoints. While higher consensus scores (e.g., seven or higher) generally indicate stronger agreement among the panelists, it is important to consider the context of the specific statement, the strength of the clinical expertise or emerging evidence, and the practical feasibility when evaluating the nutrition statements. Future research endeavors may expand our findings by exploring the impact of statements with a high consensus on the nutritional status and health outcomes of adults with chronic SCI. The researchers should also consider the emerging evidence in this field by ensuring ongoing relevance and alignment with the evolving literature.

It is also essential to acknowledge certain limitations. First, the researchers’ perspectives on the literature selection and interpretation are a factor to consider. Also, the quality and scope of the available literature can influence the strength of the nutrition recommendations generated in this study. Although the real-time Delphi methodology provided a simultaneous calculation and feedback, it still prevented a live discussion among the panelists. The Delphi method relied on the opinions of the experts [218]. The perspectives represented by the panelists may not cover the entire spectrum of professionals involved in SCI and nutritional care. Therefore, the generalizability of the recommendations should be considered within the context of the Delphi panel’s composition. In this study, we focused on individuals with chronic SCI without categorizing the literature by injury level and severity. While we recognize that the physiological needs of individuals at different stages of spinal injury may vary, we have decided to maintain a broader focus due to the limited and fragmented evidence available in the SCI nutrition literature. Similarly, some of the statements, such as the use of BMI and waist circumference cutoffs, were supported by limited or weak evidence, which requires further empirical validation in large, SCI-specific cohorts. Notably, some studies referenced in our research primarily involved male participants or specific populations, such as East Asians. To enhance the generalizability of these recommendations, future research should prioritize broader and diverse cohorts. Lastly, we acknowledge that nutritional strategies are tied to long-term adherence and ongoing engagement. While we offer cautious guidance on supplementation, the long-term impacts remain unexamined, a notable limitation of our study and the existing literature. Further work is critical to explore the long-term outcomes and strategies to enhance patient adherence to supplementation regimens.

## 5. Conclusions

This Delphi study synthesized the available evidence on the nutritional needs of adults with chronic SCI and established an expert consensus on key nutrition statements. Out of the 18 statements evaluated, 15 reached moderate agreement, and 3 achieved high agreement. Areas of moderate agreement highlight priorities for further research and validation. The expert-rated statements contribute to the ongoing dialogue in the field of SCI-specific nutrition, providing a practical and consensus-based resource for healthcare professionals. Continued research and expert collaboration are needed to enhance and implement these recommendations in practice.

## Figures and Tables

**Table 1:** Nutrition statements extracted from the literature review.

Weight and Body Composition
1- Persons with paraplegia should weigh 5–10% less than indicated in the Metropolitan Life Height–Weight Tables. Persons with tetraplegia should weigh 10–15% less than indicated in the Metropolitan Life Insurance desirable weight table.
2- Persons with spinal cord injury (SCI) whose body mass index (BMI) is 22 kg/m^2^ or greater should be considered obese.
3- Waist circumference: optimal cutoff should be 86.5 cm in men.
Energy Expenditure
4- When applicable, the SCI-specific BMR equation proposed by Chun et al. [28] should be used (Male/Female = 24.5 × FatFreeMass + 244.4).
5- In the absence of fat-free-mass information, the Mifflin–St Jeor equation should be used to predict the resting metabolic rate (RMR) in persons with chronic SCI.
6- In the SCI population, the total daily energy expenditure (TDEE) should be estimated by multiplying the RMR with the common activity correction factor of 1.15. TDEE: RMR X 1.15
Macronutrient Intake
7- Carbohydrate intake should be around 45% of total daily energy intake for people with SCI.
8- Total dietary fiber intake should be 15 to 20 g a day, with adequate fluid intake, for people with SCI.
9- Individuals with SCI need 0.8 to 1.0 g of protein per kg of body weight, or roughly 10–30% of the daily caloric intake, to maintain protein stores in the absence of pressure injuries or infection.
10- Protein recommendations for individuals with SCI who have pressure injuries are as follows: Stage II: 1.2 to 1.5 g of protein per kg body weight/per day. Stage III and IV: 1.5 to 2.0 g of protein per kg body weight/per day.
11- People with SCI should not consume more than 30% of total fat and 5–6% of saturated fat in their daily caloric intake.
Micronutrient Intake and Supplementation
12- People with SCI must consume adequate micronutrients based on the Dietary Guidelines for Americans (DGA).
13- For individuals with SCI who have developed a pressure injury, the dietitian should recommend daily mineral and vitamin supplements within the recommended dietary allowance (RDA) levels.
14- People with SCI should be prescribed dietary supplements with caution and only if certain deficiencies are detected or to prevent them.
Fluid and Sodium Intake
15- Fluid intake should be adjusted based on health condition, age, sex, climate, and physical activity level.
16- Sodium consumption should be ≤2400 mg/d for people with SCI.
Alcohol Consumption
17- Alcoholic beverages should be avoided by individuals with SCI.
Dietary Patterns
18- The DASH or the Mediterranean diet can be adopted by individuals with SCI if hypertension or other cardiometabolic risk factors are present.

**Table 2:** Demographic characteristics of the panelists.

	Total (n = 15)	RDs (n = 7)	Physicians (n = 2)	Researchers (n = 6)
Level of education N (%)				
Doctorate or professional degree	9 (60%)	1 (14%)	2 (100%)	6 (100%)
Bachelor	3 (20%)	3 (42.9%)		
Master’s degree	3 (20%)	3 (42.9%)		
Area of expertise N (%)				
SCI	12 (80%)	5 (71.4%)	2 (100%)	5 (83.3%)
Clinical nutrition	2 (13.3%)	2 (28.6%)		
Public health	1 (6.7%)			1 (16.7%)
Years of experience in the field N (%)				
1–5 years	4 (27.7%)	3 (42.9%)	1 (50%)	
6–10 years	3 (20%)			3 (50%)
11–15 years	5 (33.3%)	3 (42.9%)	1 (50%)	1 (16.7%)
16–20 years	1 (6.7%)			1 (16.7%)
21–30 years	2 (13.3%)	1 (14.3%)		1 (16.7%)
Previously involved in a Delphi study N (%)				
Yes	4 (26.7%)			4 (66.7%)
No	11 (73.3%)	7 (100%)	2 (100%)	2 (33.3%)
Familiarity with the literature (1–10)				
Mean (SD)	7.26 (1.75)	7 (1.41)	5.5 (3.54)	8.16 (1.17)
Median (IQR)	8 (1)	8 (2)	5.5 (2.5)	8 (1.5)

**Table 3:** The Metropolitan Life Height-Weight Table for calculating body frame size.

Men Height	Wrist Circumference	Frame Size	Women Height	Wrist Circumference	Frame Size
			Under 5′2″	Less than 5.5″ 5.5″ to 5.75″ Over 5.75″	Small Medium Large
			5′2″ to 5′5″	Less than 6″ 6″ to 6.25″ Over 6.25″	Small Medium Large
Over 5′5”	5.5″ to 6.5″ 6.5″ to 7.5″ Over 7.5″	Small Medium Large	Over 5′5″	Less than 6.25″ 6.25″ to 6.5″ Over 6.5″	Small Medium Large

**Table 4:** Prediction equations for estimating resting or basal metabolic rate. Adapted from Farkas et al. [61].

Equation Name/Author(s)	Year	Sex	Metabolic Rate Prediction Equation
Harris–Benedict [67,81]	1919	M	BMR = 66.4730 + (13.7516 × wt) + (5.0033 × ht) − (6.7550 × age)
		F	BMR = (1.8496 × ht) + (9.5634 × wt) + 655.0955 − (4.6756 × age)
Cunningham [72]	1980	M/F	BMR = 500 + 22 (LBM)
Schofield [68]	1985	M	BMR = 15.057 × wt + 692.2 (age, 18−30 y), 11.472 × wt + 873.1 (age, 30−60 y), 11.711 × wt + 587.7 (age, >60 y)
		F	BMR = 14.818 × wt + 486.6 (age, 18−30 y), 8.126 × wt + 845.6 (age, 30−60 y), 9.082 × wt + 658.5 (age, >60 y)
Owen [74]	1987	M	RMR = 290 + 22.3 (LBM)
		F	RMR = 334 + 1.97 (LBM)
Mifflin–St. Jeor [73]	1990	M	BMR = (9.99 × wt) + (6.25 × ht) − (4.92 × age) + 5
		F	BMR = (10 × wt) + (6.25 × ht) − (5 × age) − 161
SCI-Specific Equations			
Buchholz et al. [82]	2003	M/F	RMR = 10,682 − 1238 × (age) − 521 × (sex) − 24 × (ht) + 87 × (FFM)
Chun et al. [28]	2017	M/F	BMR= 24.5 × FFM + 244.4
Nightingale and Gorgey et al. [66]	2018	M	BMR= 23.469 × FFM + 294.330 (FFM alone)
		M	BMR = 23.995 × FFM + 6.189 × SAD + 6.384 × TAD − 6.948 × TC + 275.211 (FFM with circumferences and diameters)
		M	BMR = 19.789 × FFM + 5.156 × wt + 8.090 × ht − 15.301 × CC − 860.546 (FFM with anthropometrics)
		M	BMR = 13.202 × ht + 11.329 × wt − 16.729 × TAD − 1185.445 (anthropometrics alone)

Note. BMR = basal metabolic rate; RMR = resting metabolic rate; M = male; F = female; wt = weight (kg); K = constant for metabolic rate of organ/tissue at resting state; AT = adipose tissue; SM = skeletal muscle; RM = residual mass; ht = height (cm); FFM = fat-free mass (kg); FM = fat mass; LBM = lean body mass; SCI = spinal cord injury; SAD = sagittal abdominal diameter (cm); TAD = transverse abdominal diameter (cm); TC = thigh circumference (cm); CC = calf circumference (cm).

**Table 5:** Summary of expert ratings and consensus details for each nutrition statement.

Statements	Consensus *	Score	Median (IQR, min–max)	Responses	Stability **
Weight and Body Composition					
1- Persons with paraplegia should weigh 5–10% less than indicated in the Metropolitan Life Height–Weight Tables. Persons with tetraplegia should weigh 10–15% less than indicated in the Metropolitan Life Insurance desirable weight table.	4	4.14	5 (3, 1–8)	14	50.93%
2- Persons with spinal cord injury (SCI) whose body mass index (BMI) is 22 kg/m^2^ or greater should be considered obese.	5	5.47	6 (6, 1–9)	15	48.79%
3- Waist circumference: optimal cutoff should be 86.5 cm in men.	5	5.20	5 (3, 1–9)	15	55.67%
Energy Expenditure					
4- When applicable, the SCI-specific BMR equation proposed by Chun et al. [28] should be used (Male/Female = 24.5 × FatFreeMass + 244.4).	5	5.07	5 (3, 1–8)	15	58.15%
5- In the absence of fat-free mass information, the Mifflin–St Jeor equation should be used to predict the resting metabolic rate (RMR) in persons with chronic SCI.	4	4.40	5 (3, 1–7)	15	56.37%
6- In the SCI population, the total daily energy expenditure (TDEE) should be estimated by multiplying the RMR with the common activity correction factor of 1.15. TDEE: RMR X 1.15	7	6.60	7 (3, 2–9)	15	69.81%
Macronutrient Intake					
7- Carbohydrate intake should be around 45% of total daily energy intake for people with SCI.	6	5.73	6 (3, 1–8)	15	60.19%
8- Total dietary fiber intake should be 15 to 20 g a day, with adequate fluid intake, for people with SCI.	6	6.47	7 (3, 3–8)	15	76.72%
9- Individuals with SCI need 0.8 to 1.0 g of protein per kg of body weight, or roughly 10–30% of daily caloric intake, to maintain protein stores in the absence of pressure injuries or infection.	5	5.40	6 (4, 1–9)	15	58.71%
10- Protein recommendations for individuals with SCI who have pressure injuries: Stage II: 1.2 to 1.5 g of protein per kg body weight/per day. Stage III and IV: 1.5 to 2.0 g of protein per kg body weight/per day.	7	6.87	8 (4, 1–9)	15	60.74%
11- People with SCI should not consume more than 30% of total fat and 5–6% of saturated fat in their daily caloric intake.	6	5.93	6 (3, 1–8)	15	64.27%
Micronutrient Intake and Supplementation					
12- People with SCI must consume adequate micronutrients based on the Dietary Guidelines for Americans (DGA).	7	6.67	7 (4, 1–9)	15	61.69%
13- For individuals with SCI who have developed a pressure injury, the dietitian should recommend daily mineral and vitamin supplements within recommended dietary allowance (RDA) levels.	7	7.33	8 (3, 5–9)	15	80.26%
14- People with SCI should be prescribed dietary supplements with caution and only if certain deficiencies are detected or to prevent them.	8	8.13	9 (2, 6–9)	15	86.16%
Fluid and Sodium Intake					
15- Fluid intake should be adjusted based on health condition, age, sex, climate, and physical activity level.	8	7.73	8 (2, 6–9)	15	87.57%
16- Sodium consumption should be ≤2400 mg/d for people with SCI.	6	6.47	7 (2, 1–9)	15	62.18%
Alcohol Consumption					
17- Alcoholic beverages should be avoided by individuals with SCI.	6	5.80	6 (3, 1–9)	15	61.89%
Dietary Patterns					
18- The DASH or the Mediterranean diet can be adopted by individuals with SCI if hypertension or other cardiometabolic risk factors are present.	7	6.93	7 (3, 5–9)	15	77.88%

* Ratings of 1–3 were considered poor agreement, 4–6 moderate, and 7–9 high. ** Stability threshold is 50.00%.

**Table 6:** Expert comments and corresponding ratings for each nutrition statement.

Statements	Comments from Panelists	Consensus for the Comment	Response from the Panelists
1- Persons with paraplegia should weigh 5–10% less than indicated in the Metropolitan Life Height–Weight Tables. Persons with tetraplegia should weigh 10–15% less than indicated in the Metropolitan Life Insurance desirable weight table.	➢ WC is not a validated tool in SCI.	7	13
	➢ The Metropolitan Life table is not meant for persons with SCI and needs to be used with caution, considering the major changes in body composition after SCI.	8	14
2- Persons with spinal cord injury (SCI) whose body mass index (BMI) is 22 kg/m^2^ or greater should be considered obese.	➢ Laughton et al. [39] found among 77 individuals with SCI that using the BMI obesity cutoff of 30 kg/m^2^ failed to identify nearly 74% of the participants with obesity. Based on percentage of fat mass and c-reactive protein, the authors proposed that	7	9
	➢ the obesity classification should be between 22.1 and 26.5 kg/m^2^ for those with SCI.	6	11
	➢ Inayama et al. [214] examined the visceral fat area and suggested a BMI cutoff for obesity of 22.5 kg/m^2^.	6	11
	➢ Using the percentage of body fat as a reference, lower BMI cutoffs than the standard 22.5 kg/m^2^ have been proposed for defining obesity in the SCI population.	6	11
	➢ Tissue redistribution, edema, and level of SCI vary between individuals with SCI and likely skew BMI/WC in ways that are unable to reflect weight classification. May be interesting to look at the use of skin fold assessments.	6	11
	➢ This is well confirmed based on a %fat mass of greater than 20%	5	9
3- Waist circumference: optimal cutoff should be 86.5 cm in men.	➢ WC should be measured both in the supine AND seated position.	7	11
	➢ The statement is written for SCI—the paper by Sumrell et al. was only in men. WC in the population without SCI is broken down by gender.	8	11
	➢ The Sumrell et al. [56] paper uses a VAT cutoff of 100 cm^2^ and states, “Where significant correlations were observed for VATCSA with biomarkers of cardiometabolic disease, participants were dichotomized into two groups using accepted cut-points for abdominal”.	7	9
	➢ Cut points and protocols have not yet been validated with a large enough sample to recommend it in a clinical setting.	8	9
	➢ Do we need to consider the impact of inadequate bowel programs?	7	8
4- When applicable, the SCI-specific BMR equation proposed by Chun et al. [28] should be used (Male/Female = 24.5 × FatFreeMass + 244.4).			
5- In the absence of fat-free mass information, the Mifflin–St Jeor equation should be used to predict metabolic rate in persons with chronic SCI.	➢ Caution should be used when interpreting the RMR from para athletes and its translation to the non-athlete population with SCI.	8	10
6- In the SCI population, the total daily energy expenditure (TDEE) should be estimated by multiplying BMR with the common activity correction factor of 1.15. TDEE: BMR × 1.15	➢ We just need to validate this equation against another method that measured TDEE. This has yet to be done.	8	12
7- Carbohydrate intake should be around 45% of total daily energy intake for people with SCI.	➢ I agree. However, prospective longitudinal trials are lacking. Please also see the work of Goldsmith et al. A strong association between carbohydrate intake and other cardiometabolic risk factors was noted.	6	8
	➢ 45% Cho intake is a sensible and likely harmless recommendation. However, I think the research on dm prevention is still building and not isolated to carb intake.	8	7
	➢ Using a percentage is sensible, because in the case of reduced energy intake, total carb intake will also proportionally reduce.	7	2
8- Total dietary fiber intake should be 15 to 20 g a day, with adequate fluid intake, for people with SCI.	➢ Fiber intake should likely be individualized for individuals based on bowel dysfunction and digestive symptoms.	8	11
	➢ Fiber intake should not just be looked at in terms of bowel function but potential benefits in other areas, such as colon cancer prevention. We should learn how to optimize these preventative strategies for the SCI population as well.	8	7
	➢ Fiber recommendations should follow the Dietary Guidelines for Americans (14 g/1000 calories consumed). Since people with SCI should consume less calories, they will also consume less fiber, appropriate to their needs.	9	1
9- Individuals with SCI need 0.8 to 1.0 g of protein per kg of body weight, or roughly 10–30% of daily caloric intake, to maintain protein stores in the absence of pressure injuries or infection.	➢ I would highly consider opening the range to 1.1 g/kg. Overall, persons with SCI have a lower protein intake, and the guidelines recommended 25%. They are usually around 18% of their daily caloric intake.	6	13
	➢ Research into anabolic resistance in persons with SCI would be helpful. Considering that many physiological systems in SCI show signs of accelerated aging, protein recommendations may indeed be opened up in the direction of the range recommended.	5	9
	➢ People with SCI consume too much protein, which puts them at risk for obesity. High quality and lean sources of protein should be emphasized, rather than encouraging an increased protein intake.	7	2
10- Protein recommendations for individuals with SCI who have pressure injuries: Stage II: 1.2 to 1.5 g of protein per kg body weight/per day. Stage III and IV: 1.5 to 2.0 g of protein per kg body weight/per day.	➢ It is not “additional protein” but rather “arginine, zinc, and vitamin C” that are needed according to Sneij et al. [215].	7	9
11- For individuals with SCI who have developed a pressure injury, the dietitian should recommend daily mineral and vitamin supplements within recommended dietary allowance (RDA) levels.	➢ A diet rich in Vitamin D should be included, or vitamin D supplements should be prescribed. The list should mention vitamin D, which is an important co-enzyme, and especially persons with pressure injuries have less exposure to sunlight.	7	12
12- People with SCI should not consume more than 30% of total fat and 5–6% of saturated fat in their daily caloric intake.	➢ <6% saturated fat will become difficult for many to achieve, especially those working on healing PI. We also need to see the results from following this protocol in this population.	6	6
13- People with SCI must consume adequate micronutrients based on the Dietary Guidelines for Americans (DGA).	➢ Several of these micronutrients are not well studied in persons with SCI. You mentioned here Vit D, which is great that that you have recognized its role.	7	13
	➢ Studies are needed to determine the amount of micronutrients needed in SCI. Should not assume DGA levels.	7	9
14- People with SCI should be prescribed dietary supplements with caution and only if certain deficiencies are detected or to prevent them.			
15- Fluid intake should be adjusted based on health condition, age, sex, climate, and physical activity level.	➢ A simple way to adjust the level of fluid intake is based on the color of urine. It is always recommended to have light yellowish color urine.	7	10
	➢ Need to be mindful of cathing.	8	10
	➢ Fluid should be balanced with fiber intake in mind as well.	8	9
16- Sodium consumption should be ≤2400 mg/d for people with SCI.			
17- Alcoholic beverages should be avoided by persons with SCI.	➢ Limit consumption may be a better phrasing.	8	9
	➢ Limit consumption because of excess kcal.	7	9
	➢ Evidence is building for all populations to avoid alcohol, with no room for “moderation”. I think as we see this more, this recommendation will be inevitable.	6	7
18- The DASH or the Mediterranean diet can be adopted by individuals with SCI if hypertension or other cardiometabolic risk factors are present.	➢ Not everyone is interested in the Mediterranean diet, and you have to provide a diet based on cultural background.	7	11
	➢ These diets can be useful; however they have not been tested in persons with SCI, and if a positive energy balance still is present are they that useful?	7	10

## Data Availability

The original contributions presented in this study are included in the article. Further inquiries can be directed to the corresponding author.

## List of Abbreviations

SCI	Spinal cord injury
RD	Registered dietitian
US	The United States
RMR	Resting metabolic rate
BMR	Basal metabolic rate
BF	Body fat
BMI	Body mass index
CVD	Cardiovascular disease
SD	Standard deviation
TDEE	Total daily energy expenditure
TEF	Thermic effect of food
MET	Metabolic equivalent of task
RDA	Recommended dietary allowance
IU	International unit
DGA	Dietary Guidelines for Americans
